# A Multidimensional Perspective on Individual Differences in Multilingual Learners’ L2 Chinese Speech Production

**DOI:** 10.3389/fpsyg.2020.00059

**Published:** 2020-02-19

**Authors:** Peijian Paul Sun, Lawrence Jun Zhang

**Affiliations:** ^1^Department of Linguistics, Zhejiang University, Hangzhou, China; ^2^Faculty of Education and Social Work, The University of Auckland, Auckland, New Zealand

**Keywords:** L2 Chinese speech production, cognition, affect, socio-culture, multilingualism

## Abstract

To date, research has been undertaken to reveal factors contributing to learners’ second/foreign language (L2) speaking and/or learning at particular points in time in separate studies from cognitive, affective, and socio-cultural perspectives as individual variables. Nonetheless, little research has concurrently investigated L2 speaking with the same cohort of learners as participants from these perspectives in a single study, to obtain comprehensive and systematic understandings of how the three dimensions of factors work together in influencing individuals’ L2 speaking. This study, utilizing Segalowitz’s (2010) L2 speech production framework as the theoretical lens, examined a group of L2 Chinese multilinguals’ perceptions toward their speech performance and production ability development, attempting to comprehensively and systematically uncover the factors influencing L2 speaking from the three perspectives mentioned above. We collected data through focus groups and semi-structured interviews from 17 advanced level L2 Chinese multilinguals. The findings of the study revealed that factors that influenced the L2 Chinese multilinguals’ speech performance and their development of such an ability included the following: (1) age of acquisition, cognitive fluency, learning styles, and speaking strategies; (2) motivation, anxiety, speaking self-efficacy, and willingness-to-communicate; (3) L2 cultural interest, L2 communities, and L2 classes; and (4) multilingualism. We conclude that the development of L2 Chinese speech production ability could be the result of the synergies gained from the cognitive, affective, and socio-cultural dimensions of L2 learning and use. Insights into L2 Chinese teachers and learners in terms of how to support and sustain the improvement of L2 Chinese speech production ability are also discussed.

## Introduction

To be able to communicate is most often the ultimate goal of learning a second/foreign language (L2). Nevertheless, most people are rarely able to speak an L2 with the same degree of, or even close to, the same level of their first languages (Segalowitz, 2010). An underlying reason may be that speaking, as a cognitively demanding skill, requires speakers to have the ability not only to use various knowledge ranging from linguistic, pragmatic, to intercultural, among other things (Hughes and Reed, 2017), but also to perform without much influence from factors such as anxiety, negative self-efficacy, and low levels of motivation (Krashen, 1982; Zhang, 2001; Ellis, 2015; Jin and Zhang, 2019). Although research has been undertaken to reveal factors contributing to learners’ L2 speaking and/or learning at particular points in time in separate studies from cognitive, affective, and socio-cultural perspectives as individual variables, little research has concurrently investigated L2 speaking from the above three perspectives with the same cohort of learners as participants in one single study in an effort to obtain more comprehensive and systematic understandings of how cognitive, affective, and socio-cultural factors work together in influencing individuals’ L2 speaking performance. In addition, much attention has been drawn to multilingual learners’ L2 learning in recent years, but there is no research so far, to our knowledge, investigating multilinguals’ perceptions toward their L2 Chinese speech performance and production ability development.

To fill such a research gap, this study, within Segalowitz’s (2010) theoretical framework on L2 speech production, comprehensively and systematically examined a group of multilinguals’ perceptions toward their L2 Chinese speech performance and production ability development. We recruited multilinguals of advanced L2 Chinese proficiency for the following reasons. First, there has been a call in place for some time for carrying out more research into the field of L2 Chinese. Second, learners with an advanced L2 proficiency often encounter an L2 learning plateau, particularly in terms of L2 speech production. Third, multilingualism has drawn its increasing attention from researchers in the field of L2 education. We hope that this empirical study could offer insights into L2 Chinese education and beyond in terms of how to support and sustain learners’ development of L2 Chinese speech production ability.

### Theoretical Framework and Related Literature

Segalowitz’s (2010) L2 speech production framework may be the most comprehensive interpretation for factors that could possibly influence L2 speech output drawing from cognitive (i.e., cognitive fluency, cognitive experiences), affective (i.e., motivation to communicate), and social dimensions. According to the framework, cognitive perceptual processing systems, motivation to communicate, interactive social contexts, and fluency-related perceptual and cognitive experiences are the four prominent components contributing to learners’ L2 speech production (see Segalowitz, 2010, for details), which are presented in some detail below.

#### Cognitive Processing System and Cognitive Dimensions of Speech Production

Cognitive perceptual processing systems in Segalowitz’s (2010) L2 speech production framework are basically an integration of Levelt’s (1989) and de Bot’s (1992) speech production models, showing that the production of overt speech involves planning, encoding, and articulation procedures (see Segalowitz, 2010). According to Segalowitz (2010), cognitive processing systems could directly influence individuals’ L2 speech production. Such a position resonates with previous studies suggesting that L2 speech production can be subject to influences of cognitive factors such as working memory, speech planning, and cognitive fluency (e.g., Kahng, 2014; Cho, 2018; Li and Fu, 2018). Cognitive fluency, as a major indicator of any cognitive processing system, refers to “the efficiency of operation of the underlying processes responsible for the production of utterances” (Segalowitz, 2010, p. 52). Limited research on cognitive fluency has attempted to operationalize it through measures of lexical access/retrieval speed, processing stability, attention flexibility, articulation speed, and sentence building speed (see, e.g., De Jong et al., 2013; Segalowitz, 2010).

Apart from the pioneering investigation into cognitive fluency (Kahng, 2014), how other cognitive factors such as learning styles and strategies could possibly influence multilinguals’ L2 speech performance and production ability development still remains under-researched despite the importance of learning styles and strategies that has been acknowledged in the L2 literature. Previous research on the role of learning styles has documented conflicting results, showing either positive or negative influences of different learning styles on L2 learning. For instance, Bailey et al. (2000) study of 100 university L2 learners revealed that kinesthetic style was negatively correlated with L2 achievement despite that kinesthetic style has been consistently suggested as a preferred learning style among students (e.g., Isemonger and Sheppard, 2003; Sun and Teng, 2017). Kim and Kim’s (2014) study of 2,682 Korean L2 English learners found that visual style was not only positively correlated with L2 proficiency but also the most influential variable in affecting learners’ L2 advancement. With regard to learning strategies, a growing body of research showed positive effects of learning strategies on learners’ self-efficacy, L2 proficiency, and learning outcomes. For example, Forbes and Fisher (2018) examined five advanced L2 French teenager learners and found that metacognitive strategies could enhance learners’ confidence and proficiency in speaking. Teng and Zhang’s (2018) study of 512 L2 English learners’ motivational regulation strategies revealed that such strategies could both directly and indirectly (mediated through cognitive and metacognitive strategies) contribute to L2 writing production performance.

In addition to the above cognitive dimensions pertaining to speech production, age of acquisition is another critical factor that has been broadly examined for its impact on L2 learning, development, and attainment (Hartshorne et al., 2018). To be specific, age of acquisition has been tightly associated with L2 speech perception and production. For instance, it was suggested that early L2 learners, compared with late L2 learners, tend to be more native-like at grammatical, lexical, phonological, and prosodic levels (Archila-Suerte et al., 2015; Saito, 2015). Overall, the effect of age on acquisition has been widely evidenced in the fact that late L2 learners “appear to be less successful in acquiring a L2,” at least at phonetic and phonological levels (Lahmann et al., 2016, p. 357).

#### Motivation to Communicate and Affective Dimensions of Speech Production

Motivation to communicate, as an affective dimension of Segalowitz’s (2010) L2 speech production framework, refers to learners’ socially grounded beliefs about communication. According to Segalowitz (2010), motivation to communicate has both direct and indirect (mediated by social context) influences on L2 speech production because it supports learners’ engagement in L2 communication. Such a position echoes the well-established motivation literature of the positive effects of motivation on the success of L2 learning (Hernández, 2006; Shaikholeslami and Khayyer, 2006). For example, Hernández’s (2010) study of 20 L2 Spanish learners’ oral proficiency revealed that motivation could determine learners’ interaction with Spanish culture, which in turn could help the improvement of their speaking in Spanish.

In line with motivation to communicate, MacIntyre et al. (1998) L2 willingness to communicate (WTC) model offers a more comprehensive understanding of L2 use, in which two underlying variables directly account for WTC, namely the desire to communicate with a specific person and the state of one’s communicative self-confidence. Since the conceptualization of the L2 WTC model, WTC as a theoretical construct has gained popularity among researchers given that communicative interaction is believed to be indispensable for L2 development (Kang, 2005). For example, Amiryousefi’s (2018) investigation of 612 pre-intermediate L2 English learners’ WTC revealed that motives to communicate with teachers and motivational interests could significantly enhance students’ L2 WTC and speech performance.

Although a considerable number of studies have examined the impact of various motivations (i.e., integrated, intrinsic, and extrinsic motives, and WTC) on L2 learning and use, the influences of other affective factors such as anxiety and self-efficacy on L2 speech production also worth mentioning. In effect, anxiety and self-efficacy have been long recognized as prime factors determining individuals’ L2 learning and use. For instance, Teimouri et al. (2019) meta-analysis of 105 studies with a total number of 19,933 from 23 countries confirmed that anxiety played a negative role in L2 learning and use, particularly listening and speaking performance. More recently, scholars have argued for understanding L2 learning from the perspective of positive psychology (Dewaele and Li, 2018; Li et al., 2018; Dewaele, 2019; Jin and Zhang, 2019). Such a view indicates that affect does really have an important role to play in accounting for L2 learning success, especially speech production.

Related to the concept of positive psychology is the notion of self-efficacy, which forms part of the learners’ perceptions of how to achieve success (Teng and Zhang, 2018; Chen and Zhang, 2019). Sardegna et al. (2018) study of 704 L2 English learners’ pronunciation learning revealed that self-efficacy could influence learners’ learning strategy choices, which consequently could contribute to their pronunciation improvement. In addition to the respective influence of anxiety and self-efficacy on L2 learning and use, recent research shows that the two factors could jointly determine learners’ L2 performance. For example, Phongsa et al. (2018) study of 242 L2 English college students suggested that high self-efficacy reduced anxiety (e.g., fear of ambiguity), which in turn enhanced learners’ L2 gains and performance (Horwitz, 2001).

#### Environment, Experience, and Socio-Cultural Dimensions of Speech Production

The social dimension of Segalowitz’s (2010) L2 speech production framework lies in its emphasis of the influence of the environment and experience on L2 speech production. According to Segalowitz (2010), social context is where L2 communicative experience is embedded, and such experience in turn could shape the development of individuals’ L2 speech production ability. In response to the importance of experience and the environment in L2 learning and use, together with the fact that culture is an indispensable contextual affordance of society, a growing number of studies have been carried out from the socio-cultural perspective, particularly in the study-abroad context (Taguchi and Li, 2019).

One line of such research has qualitatively examined how learners’ interaction in the study-abroad context contributes to their L2 development (e.g., enlarged vocabulary, improved communicative skills, and enhanced intercultural competence). For example, Shively (2011) observed seven L2 Spanish learners’ pragmatic development in making requests, suggesting that socialization in the study-abroad environment could help the learners adopt appropriate verbs to make requests. In contrast, DeKeyser’s (2010) investigation of 16 L2 Spanish learners found that the benefits of studying abroad could not be guaranteed if the input and interaction were beyond learners’ current linguistic knowledge.

Another line of research into the influence of studying abroad on L2 development, on the other hand, was explored through quantitative methods. Segalowitz and Freed (2004), for example, examined 40 L2 Spanish learners’ L2 speech gains in home university and study-abroad settings. The findings showed that learners had greater L2 speech performance gains in the study-abroad context. In a similar vein, Yashima and Zenuk-Nishide’s (2008) investigation of 165 L2 English learners revealed that the study-abroad experience provided learners with more language contact, resulting in more gains in their L2 speech performance and production ability. In order to add more robust evidence statistically, Taguchi et al. (2016) adopted latent growth curve modeling to examine the effects of intercultural competence and social contact on learners’ gains in terms of their L2 Chinese speech production. It was found that the amount of social contact (i.e., L2 use and social involvement) could directly account for learners’ improvement in L2 Chinese speech acts such as refusals and requests.

Apart from the contextual influence, the cultural impact, particularly through the lens of culture interest, on L2 learning and speaking has also been reported. For example, Nomura and Yuan’s (2019) study of 30 L2 Japanese learners’ learning motivations found that cultural interest was the major motivation for learning Japanese. Minagawa et al. (2019) study of 348 L2 Japanese learners further evidenced that cultural interest was one of the two top reasons for L2 Japanese learning. Similar findings could also be found in terms of the effect of cultural interest on L2 speaking. For instance, Amiryousefi’s (2018) study of 612 L2 English learners revealed that interest could significantly contribute to learners’ willingness to communicate and speak.

#### Multilingualism and Bilingualism

Multilingualism and bilingualism are two generic terms referring to the phenomenon of speaking and understanding two or more languages. Bilingualism, in a narrow sense, includes two languages (de Groot, 2011), and in a broad sense, two or more languages (Cook and Bassetti, 2011). Multilingualism refers to situations where two or more languages are involved, either in language learning and/or acquisition or in language use (Aronin and Singleton, 2008). In general, multilingualism can be regarded as “a generic term including bilingualism” (Cenoz, 2013, p. 7).

Multilingualism has drawn increasing attention in recent years not only due to the fact that “multilingualism is as pervasive in the world today as it has always been” (Ortega, 2019, p. 24) but also because of the existence of distinct processing mechanisms of multilingual learners (Higby et al., 2013). Researchers have examined multilingualism from various perspectives. Much research has focused on the cognitive dimension of multilingualism, pointing out that there are cognitive advantages of multilinguals over monolinguals in terms of working memory, perception, and attentional and inhibitory control (Bright et al., 2019). Additionally, researchers have also examined multilingualism from affective and social dimensions, including factors such as emotion, affect, social contact, and social context, due to the fact that language learning is an emotionally driven and socially engaging process (Cenoz and Gorter, 2019).

#### Summing up

It can be drawn from the above theoretical framework and literature review that L2 speech performance and production ability can be subject to cognitive (i.e., age of acquisition, cognitive fluency, learning styles, and learning strategies), affective (i.e., WTC, motivation, anxiety, and self-efficacy), and socio-social (i.e., learning context, social contact, and cultural interest) factors. Nonetheless, how the three dimensions of factors concurrently influence L2 speaking with a particular focus on L2 Chinese still remains under-researched. Drawing on Segalowitz’s (2010) L2 speech production framework, this study aims to establish a comprehensive and systematic understanding of multilinguals’ perceptions toward their L2 Chinese speech performance and production ability development from cognitive, affective, and socio-cultural perspectives. It is hoped that insightful suggestions could be provided for teachers and learners in terms of the improvement of multilinguals’ L2 Chinese speech production ability, particularly at the advanced level. This study was guided by the following overarching question: *How do cognitive, affective, and socio-cultural factors contribute to multilinguals’ L2 Chinese speech performance and production ability development*?

## Materials and Methods

### Participants

A total of 17 multilinguals with an advanced level of L2 Chinese proficiency were recruited through convenient and purposive sampling in order to ensure that participants share a similar L2 Chinese learning background (see Table 1, for details). The 17 voluntary multilinguals were undergraduates or postgraduates from two universities in Beijing, China. They all majored in L2 Chinese Education but with different orientations (i.e., Business Chinese, Chinese Literature, and L2 Chinese Teaching). A common feature of the participants was that they had another L2 or L2s learning experience in addition to their L2 Chinese learning in China currently. They all reported that Chinese was their dominant L2, except for Bai and Steve, who claimed that Russian and English were more native languages to them, respectively. The 17 participants’ average age was 23.65 (*SD* = 4.55). They all had 3–5 years of classroom Chinese learning experience in their home countries and had been learning Chinese in China for more than 2 years prior to this study. All the participants were given pseudonyms for the sake of anonymity and confidentiality. Focus group participants were particularly asked to respect one another’s privacy, given that anonymity could not be guaranteed within group discussions.

**TABLE 1:** Background information of L2 Chinese multilinguals.

**Name**	**L1**	**L2s**	**Age**	**Gender**	**Nationality**	**Interview**
Lin	Spanish	Chinese and English	18	Female	Panama	FG
Bai	Mongolian	Russian, Chinese, and English	26	Female	Mongolia	FG
Yeats	English	Chinese and Irish	21	Male	USA	FG
Tao	Thai	Chinese and English	25	Female	Thailand	FG
June	Burmese	Chinese and English	26	Female	Burma	FG
Mia	Burmese	Chinese and English	25	Female	Burma	FG
Krimu	Russian	Chinese and English	25	Male	Russia	FG
Feng	Burmese	Chinese and English	25	Female	Burma	FG
Cai	Thai	Chinese and English	27	Male	Thailand	FG
Hanna	Korean	Chinese and English	18	Female	Korea	FG
Judy	Korean	Chinese and English	18	Female	Korea	FG
Jenny	Korean	Chinese and English	18	Female	Korea	FG
Dan	English	Chinese and French	22	Male	USA	Individual
Mads	Norwegian	Chinese and English	33	Male	Norway	Individual
Steve	German	English, Chinese, and Italian	31	Male	Austria	Individual
Gaoen	Korean	Chinese and English	19	Female	Korea	Individual
Tom	English	Chinese and Japanese	25	Male	USA	Individual

*FG, focus group; L2, second/foreign language.*

### Instruments

Two types of interviews, namely, focus groups and semi-structured interviews (see Appendix), were adopted to bring different lines of sight into analysis so that a profound and appropriate understanding of a phenomenon could be ensured (Patton, 2015). Specifically, the two types of interviews were employed to collect the qualitative data from collective and individual perspectives respectively in order to solicit multilinguals’ perceptions toward factors contributing to their L2 Chinese speech performance and production ability development. One-on-one interviews were adopted given that the environment of such interviews was securer and more confidential compared with that of focus groups. As a result, as scholars have argued, interviewees may be more open to share their deep feelings. Also, different from one-on-one interviews, focus groups offer participants opportunities to agree or disagree with the comments of others. Consequently, this may add richness to the dialogue (Tavakoli, 2012).

Prior to the data collection, the two types of interviews were piloted with six multilinguals with the advanced level of L2 Chinese. The main purpose of piloting was to ensure the practicality of the interview questions. One major issue that occurred during the pilot was that the participants found the terminology such as *kinesthetic* and *tactile* rather abstract. A brief explanation was, therefore, added to the actual interviews to help participants’ better understanding of the terminology. Visual assistance such as pictures was also provided for facilitating the discussion.

### Data Collection and Analysis

The data collection lasted 4 months. Prior to the data collection, the first author circulated the participant recruitment advertisement among three universities in Beijing and made initial contacts with the L2 Chinese learners who expressed their willingness to participate. A total of five focus groups were conducted with 18 participants for eliciting their collective opinions on L2 Chinese speech performance and the development of such an ability. In each group, three to five learners participated in an approximately hour-long discussion. Please note that only 12 participants from focus groups were reported in this study, as they were self-diagnosed as multilinguals with the advanced level of L2 Chinese proficiency. Apart from focus groups, five semi-structured interviews (40–60 min/each) were carried out for the elicitation of multilinguals’ opinions on L2 Chinese speech performance and production ability development from an individual level. In total, 17 multilingual L2 Chinese learners from two universities were documented.

The collected qualitative data were then transcribed verbatim by the first author. After transcription, codes were attached to sentences or whole paragraphs in order to dissect them meaningfully (Miles et al., 2013). Consequently, a coding system was developed through searching for regularities, patterns, or topics in the data, writing down words and phrases that represent these topics and patterns, and developing a list of coding categories (Bogdan and Biklen, 2007). After initial coding, the first author went through the created coding system for refinement so that there were no overlaps or redundancies in the coding categories. It was then checked by the second author. The whole process of coding and categorizing was accomplished by using NVivo 10 software. When reporting results, the quotes from the participants were translated from Chinese to English with redundant information deleted.

In this study, confirmability was achieved through repeated coding of the data. A Ph.D. candidate in Chinese applied linguistics was invited to be a peer debriefer. A random sample of 20% of recording transcription was taken for recoding with reference to the developed coding system. All the codes were then numbered and imported into SPSS for the inter-rater reliability test. The results of a paired-samples *t*-test indicated that the two raters’ codes were highly consistent (*r* = 0.96, *p* = 0.001).

## Results and Discussion

### Cognitive Factors Influencing Multilinguals’ L2 Chinese Speech Performance and Production Ability Development

This study revealed that age of acquisition, cognitive fluency, learning styles, and speaking strategies were main cognitive factors contributing to the multilinguals’ L2 Chinese speech performance and production ability development (see Table 2).

**TABLE 2:** Cognitive dimensions of multilinguals’ L2 Chinese speech production.

**Cognitive factors**	**Views on contributing factors to L2 speech production**
Age of acquisition	∙ Positively linked with L2 speech production ability and performance (14/17) ∙ Practice is essential (3/17)
Cognitive fluency	∙ Positively linked with L2 speech production performance (13/17)
Learning styles	∙ Group style provides adequate L2 output opportunities, thus positively contributes to L2 speech production ability development (8/17) ∙ Audio/visual style improves learners’ sense of speech authenticity, which in turn strengthens their speech confidence and consequently their L2 speech production ability (5/17) ∙ Individual style provides a more comfortable and self-regulated learning environment for leaner’s to improve their L2 speech production ability (4/17)
Speaking strategies	∙ Practice-oriented strategy is positively related with L2 speech production ability development (10/17) ∙ Substitution-oriented strategy is positively related with L2 speech production performance and the development of such an ability (7/17)

#### Age of Acquisition

Fourteen out of the 17 multilinguals suggested that the onset of L2 Chinese learning (age of acquisition) could be the reason distinguishing each other’s L2 speech performance and production ability. As Gaoen pointed out, “I do believe that the longer you learn, the better your Chinese will become, such as larger vocabulary, better pronunciation, and more nativelike. I think a person’s speaking ability and performance can be tightly correlated with when he/she starts learning an L2” (Individual, Gaoen 27/06/2014). Three out of the 17 multilinguals, despite agreeing on the importance of the age of acquisition, pointed out that speech production was a skill that, to a large extent, depended on the frequency of practice. For example, “if I started learning another language at an early age but without much practice, I think I still could not speak well” [focus group (FG), Feng 08/06/2014].

Corroborating the critical period hypothesis that a language could not be fully acquired after puberty, our data also showed that age of acquisition has a critical impact on determining learners’ L2 speech production ability (Hartshorne et al., 2018). However, different from previous research claiming that late L2 learners may not be able to become native-like especially in terms of their acquisition of acoustic features (e.g., Saito, 2015; Lahmann et al., 2016), our results showed that age of acquisition might weaken its impact on L2 speech development without the joint effort of frequent communicative output. In other words, age of acquisition alone could not fully determine the outcome of learners’ L2 speech production ability. Given that there is little solid evidence showing that adults are inferior learners (DeKeyser, 2013), teachers and learners should understand that what matters may not be the onset age of L2 learning but the quality of L2 teaching, the frequency of L2 practice, and the active engagement of L2 learning, which jointly determine learners’ L2 speech performance and production ability development.

#### Cognitive Fluency

Cognitive fluency, according to Segalowitz (2010), can be understood as individuals’ efficiency or automaticity of cognitive process in speaking. Thirteen out of the 17 multilinguals suggested that cognitive fluency (processing speed) could positively contribute to their L2 Chinese speech performance. As Dan said, “I think processing speed has a lot to do with my speaking performance. I cannot speak fast, because I have to organize in my mind first before speaking. I think processing speed is very important. It represents how good your speaking ability is” (Individual, Dan 16/05/2014). Steve also claimed, “I think the faster you organize or process what you want to say the better your speaking ability is. They are positively correlated” (Individual, Steve 23/06/2014).

Comparing with my (L2) Russian, my Chinese is much slower. I guess because I grew up in a bilingual environment, my Russian is more like a mother tongue to me. If I grew up in a Mongolian and Chinese bilingual environment, I would speak Chinese much faster than my other foreign languages. I think cognitive fluency can determine how fast you speak and it has a lot to do with environment (FG, Bai 10/06/2014).

As suggested, cognitive fluency can be a direct reflection of the multilinguals’ L2 Chinese speech performance and production ability. Such a result adds evidence to Segalowitz’s (2010) L2 speech production model, positing that learners’ cognitive processing system could directly influence their L2 speech production. Therefore, we suggest that teachers design relevant classroom activities to improve learners’ L2 automaticity or cognitive processing ability. For example, scaffolding activities might contribute to the cognitive fluency development of the novice L2 learners, while impromptu speech might be beneficial for the advanced L2 learners. As for learners, they may consider practicing cognitively demanding tasks to push the development of their automaticity in L2 speech production.

#### Learning Styles

All the 17 multilinguals suggested that there was a positive link between learning styles and their L2 Chinese speech production ability development, particularly group, auditory/visual, and individual learning styles. To be more specific, 8 out of 17 multilinguals claimed that the group learning style was of great importance for the improvement of their L2 Chinese speech production ability. As Tao pointed out, “compared with individual learning, group learning provides you with more opportunities to output. Such output is the foundation for the improvement of a person’s speaking ability” (FG, Tao 12/04/2014). Mads also mentioned that “learning by yourself without socializing with others, you can hardly make any progress in speaking” (Individual, Mads 23/06/2014). The perceived advantage of group learning, according to the participants, lies in the fact that such a style compared with other learning styles provides more meaningful and interactive opportunities for learners to verbalize their ideas and knowledge, which in turn may lead to more gains in L2 speech production ability development.

Regarding the auditory/visual learning style, 5 out of the 17 multilinguals claimed that this style could be a factor that directly contributes to their L2 Chinese speech production ability development. As Gaoen mentioned, “watching Chinese television is an effective way to improve my spoken Chinese. By listening and watching to the authentic Chinese, I developed a sense of authenticity which contributes not only to my confidence in speaking but also to the development of my speaking ability” (Individual, Gaoen 27/06/2014).

In terms of the individual learning style, 4 out of the 17 multilinguals claimed that they preferred studying Chinese individually, as individual learning provided them with a much safer, less disturbing, and more self-regulated environment to enhance the development of their L2 speech production ability. For instance, “studying alone is more efficient for me. When studying in groups, I will easily get distracted and end up gossiping with my Korean peers. Studying alone does not mean you cannot practice speaking; you can do self-talking” (FG, Jenny 10/06/2014).

The above findings revealed that group, auditory/visual, and individual styles are the three main learning styles that the multilingual L2 Chinese learners utilize to support the development of their L2 speech production ability. However, such a result does not show much support to the literature that learners favor a kinesthetic style and disfavor group and individual styles (Isemonger and Sheppard, 2003; Naserieh and Sarab, 2013; Sun and Teng, 2017). We suggest that teachers not overtailor their classes according to students’ learning styles but rather be more attentive to the improvement of their classroom teaching efficiency. Learners, on their own, should take advantage of their dominant or preferred learning style to improve their L2 speech production ability. Learners should also consider stepping out of their comfort zone by utilizing other learning styles that may possibly contribute to the development of L2 speech production ability.

#### Speaking Strategies

Practice-oriented and substitution-oriented strategies were the two major speaking strategies adopted by the 17 L2 Chinese multilinguals. They suggested that speaking strategies, particularly practice-oriented, could positively contribute to their L2 Chinese speech production performance and the development of such an ability (Sun et al., 2016).

Regarding the practice-oriented speaking strategy, 5 out of the 17 multilinguals pointed out that there was an unconscious accumulation stage before their actual speaking practice. They tried not to consciously memorize new words but, rather, unconsciously picked up some words or expressions that grabbed their attention. As June said, “when I watch TV, I try not to consciously understand every word. Rather, I try to figure out the meaning of the new words from the context. Afterward, I will practice the new words or expressions” (FG, June 12/04/2014). However, another 5 out of the 17 multilinguals’ practice-oriented speaking strategy was to consciously memorize some words and expressions before putting them into actual practice. They found that this was the best way for them to improve their L2 speech production ability. As Hanna added, “I often attentively listen to my Chinese friends’ conversations for useful words and expressions. When I have chance, I will use what I have memorized in my speaking” (FG, Hanna 10/06/2014). Although the 10 learners had different views on memorization, they all agreed that the practice-oriented speaking strategy had a positive impact on their L2 Chinese speech production ability development.

Regarding the substitution-oriented strategy, 7 out of the 17 multilinguals indicated that they preferred using such a strategy to help express their ideas. This strategy shares a similarity with the compensation strategy (Oxford, 1990) and paraphrasing (Tarone, 1981) in enabling learners to carry on communication with interlocutors through alternative ways. The respondents believed that this strategy was closely and directly related to the performance of their speech production and the development of such an ability. As Tao explained, “if you stop frequently to look for words to get your meaning crossed, you will leave an impression for others that you are not good at speaking” (FG, Tao 12/04/2014). Therefore, “if you want to speak fluently without much stop, you should take advantage of the substitution strategy” (Individual, Tom 29/06/2014).

In brief, the L2 Chinese multilinguals found it beneficial to use practice- and substitution-oriented strategies for improving their L2 speech production performance and the development of such an ability. Although such a finding enriches our understanding of the multilinguals’ L2 Chinese speaking strategies, teachers and learners should recognize that other strategies such as metacognitive strategies could also be conducive to the development of L2 speech production ability (Forbes and Fisher, 2018). In addition, teachers and learners should also realize that the development of L2 speech production ability is not merely reliant on speaking strategies but, rather, through constant and meaningful social contact, interaction, and practice by using the target language.

### Affective Factors Influencing Multilinguals’ L2 Chinese Speech Performance and Production Ability Development

This study revealed that motivation, anxiety, speaking self-efficacy, and WTC were influential affective factors influencing the multilinguals’ L2 Chinese speech production performance and the development of such an ability (see Table 3).

**TABLE 3:** Affective dimensions of multilinguals’ L2 Chinese speech production.

**Affective factors**	**Views on contributing factors to L2 speech production**
Motivation	∙ Both intrinsic (4/17) and extrinsic (13/17), including work-, introjection-, and people-driven) motivations are main impetus for the improvement of learners’ L2 Chinese speech production ability.
Anxiety	∙ Fear-related anxiety (10/17), including negative evaluation and interrupted communication flow), and test (8/17) anxieties can both facilitate and debilitate learners’ L2 Chinese speech production performance and the development of such an ability.
L2 speaking self-efficacy	∙ L2 speaking self-efficacy is positively linked with learners’ L2 Chinese speech production performance and the development of such an ability (17/17) ∙ It can be subject to the frequency of practice (17/17)
Willingness-to- communicate (WTC)	∙ WTC can positively contribute to learners’ L2 Chinese speech production performance (17/17) ∙ WTC influences speech quantity more than speech quality (5/17) ∙ WTC can be volitionally controlled (7/17)

#### Motivation

The 17 multilinguals all believed that motivation, either intrinsic, extrinsic, or integrative, provided them with the main impetus to improve their L2 Chinese speech production ability. To be more specific, 4 out of the 17 pointed out that they were mainly inspired by their intrinsic motivation. As Mads pointed out, “I am interested in communication. Therefore, I make more effort to improve my speaking ability, such as talking to Chinese friends, learning Chinese online, and visiting China” (Individual, Mads 23/06/2014). This echoes the case of Steve, who claimed, “I have always been interested in Chinese and China even when I was a child. When I was a student in Austria, there was no opportunity for me to learn Chinese. My desire to learn Chinese and speak Chinese well has been accumulating since then” (Individual, Steve 24/06/2014).

Apart from the intrinsic motivation, 13 out of the 17 multilinguals were extrinsically motivated. Three main extrinsic motivations emerged, including work-driven, introjection-driven, and people-driven motivations. Work-driven motivation was one of the most frequently mentioned extrinsic motivations. Such outward work-driven motivation often stimulated and sustained learners’ inward responsibility toward their work or their inward desire for getting the work. For example, “I was a Chinese language teacher in Thailand. I came to China for my MA degree, as I believe it is very important for my career development. Thanks to the program, my teaching and my Chinese language speaking abilities have improved much” (FG, Tao 12/04/2014).

Introjection-driven motivation refers to learners’ behavior regulation out of shame, guilt, and ego-enhancement (Ryan and Deci, 2000). For example, June (FG, June 12/04/2014) felt that she represented her country when she spoke Chinese. She did not want to leave an impression on her multinational classmates that students from Burma could not speak Chinese well. Therefore, she constantly reminded herself that she must study hard on behalf of her country. Such an introjection-driven motivation was also found in Krimu (FG, Krimu 08/06/2014), who pointed out, “I will feel ashamed of myself if I cannot speak Chinese well in front of beginners.”

People-driven motivation was another commonly mentioned extrinsic motivation. Jenny mentioned that she tried to speak good Chinese in order to make her parents feel proud of her and also to gain face for them (FG, Jenny 10/06/2014). June also added, “I want to improve my Chinese, particularly my spoken Chinese, while I am doing my MA degree here. With my knowledge gained from my MA program and my improved spoken Chinese, I am sure it will benefit my students more in Burma” (FG, June 12/04/2014).

Overall, the above findings showed that both intrinsic and extrinsic (i.e., work-driven, introjection-driven, and people-driven) motivations sustained the improvement of the multilinguals’ L2 Chinese speech production ability. Such a result corroborates the literature that motivation is a significant variable in influencing learners’ development of L2 speaking (Tsiplakides and Keramida, 2009; Hernández, 2010; Toni and Rostami, 2012; Akkakoson, 2016). Nevertheless, learners’ motivation construction and formation are a complex process and can be subject to different contexts (Ushioda, 2006). Therefore, teachers should not only endeavor to understand learners’ different types of motivation for the development of their L2 speech production ability but also create a more engaging and interesting classroom environment for enhancing learners’ motivation for their continuous L2 speech production ability development.

#### Anxiety

There were two main types of L2 anxiety that emerged from the participants, including fear-related and test anxiety. Specifically, 12 out of the 17 multilinguals pointed out that L2 anxiety could be both facilitating (when anxiety is not intense) and debilitating (when anxiety is intense) to their L2 Chinese speech performance and the development of such an ability. However, 5 out of the 17 did not have any L2 anxiety problem when speaking Chinese in either formal or informal contexts.

Fear-related anxiety was a salient cause for the participants’ broken L2 Chinese speech performance. Six multilinguals pointed out that their fear of being negatively evaluated and of impeding the communication flow caused their speech breakdowns. Such examples include: “I am worried when I am speaking, particularly when I speak disfluently. Others will think that you are a postgraduate student, and is this your Chinese level? I am scared of this judgement” (FG, Tao 12/04/2014); “Although I like speaking Chinese with my friends, to communicate with them, I am concerned that if I make mistakes or if I cannot speak so well, they will think that okay you are a postgraduate and you cannot speak well” (FG, Krimu 08/06/2014). Four multilinguals mentioned that they were “very scared of causing misunderstandings” (FG, Cai 08/06/2014) if there was something wrong with their expressions. Therefore, they did not want to “interrupt the normal pace of communication among friends” (FG, Feng 08/06/2014). Such a fear of impeding the communication flow hindered learners from taking initiative in communication and thus the development of their speech production ability.

Test anxiety was another type of L2 anxiety that was commonly reported by the L2 Chinese multilinguals (8/17). In fact, L2 Chinese speaking itself was not a problem for the multilinguals. It was the pressure caused by tests that, to a large extent, affected their L2 Chinese speech performance and production. As Lin pointed out, “you only have one chance for an oral exam, if you cannot get a good score, your GPA will be affected. The pressure is huge. … such anxiety and struggle will result in making more mistakes” (FG, Lin 15/05/2014).

To sum up, it was found that anxiety, such as fear-related and test anxiety, was closely related to the multilinguals’ L2 Chinese speech production and performance. Despite the negative effects of anxiety on L2 speech production (Jiang and Dewaele, 2019b), it was also suggested that anxiety could positively contribute to learners’ L2 speech performance and production ability development. This finding corroborates the literature that anxiety could be facilitating and debilitating (Zhang, 2000, 2001; Dornyei, 2005; Jin and Zhang, 2018, 2019). Teachers, therefore, should learn to maintain an appropriate level of anxiety among students in order to maximize the positive effect of anxiety on L2 speech performance and production ability development. Instead of focusing on the debilitating role of anxiety, teachers should understand that a moderate level of anxiety has its potential contributions to L2 development from a positive psychology perspective (Mercer and MacIntyre, 2014). For example, creating friendly, exciting, and engaging environments may boost students’ enjoyment of L2 learning and, subsequently, their positive emotions toward L2 speaking (Dewaele et al., 2019; Jiang and Dewaele, 2019a; Jin and Zhang, 2019).

#### Speaking Self-Efficacy

The majority of the L2 Chinese multilinguals (15/17) claimed that they were confident about their L2 Chinese speech production and performance in most cases (except in complicated and in-depth discussions), while the rest (2/17) indicated that they were not. Regardless of the perceptions of their own speaking self-efficacy, the multilinguals all agreed that there was a positive link between speaking self-efficacy and L2 Chinese speech performance and the development of such an ability. As June pointed out, “self-confidence can be closely related with speech production. If you are a confident speaker, you will not suffer much from anxiety and nervousness which can exert negative influence on your speech performance” (FG, June 12/04/2014). The participants also pointed out that their L2 speaking self-efficacy was mostly subject to the frequency of practice in addition to self-encouragement and others’ compliments. For example,

If you want to speak well, you have to be confident. There are two factors that could support the maintenance of your speaking confidence. One is external, which is the compliments from others. The other is internal. You have to say to yourself over and over again that you can do it. Without speaking self-efficacy, you cannot go far (FG, Krimu 08/06/2014).

As a whole, our study showed that there was a positive link between speaking self-efficacy and multilinguals’ L2 Chinese speech production and performance. Specifically, our study not only enriches the literature that speaking self-efficacy could positively contribute to L2 speech performance and the development of such an ability, but also adds to our knowledge of the importance of learning strategies in the enhancement of learners’ speaking self-efficacy (e.g., Sardegna et al., 2018; Zhang et al., 2019). Therefore, we suggest that teachers should strive to strengthen learners’ L2 speaking self-efficacy for the maintenance and improvement of their L2 speech performance and production ability both internally and externally. From an external side, teachers may consider organizing more regular communicative activities for learners in light of the positive influence of frequency of practice on speaking self-efficacy. From an internal perspective, teachers may consider equipping learners with anxiety reduction strategies, such as cognitive–affective talk, reflective self-talk, and positive self-talk, to boost their speaking self-efficacy (Toyama and Yamazaki, 2019).

#### WTC

WTC was reported to have a positive contribution to the multilinguals’ L2 Chinese speech performance and production ability development. Specifically, 5 out the 17 multilinguals indicated that WTC could influence speech quantity more than speech quality. For example, Dan mentioned, “If I do not want to speak to a person or I do not like the person, I may speak less. However, it does not mean that my overall speaking ability is getting worse” (Individual, Dan 15/06/2014). Interestingly, seven participants pointed out that WTC could be volitional, which means that regardless of willingness, their volition could determine how much they would like to participate in a conversation. For instance: “I used to be more willing to practice my Chinese with people whom I like. I did not talk to people like construction workers. I felt we were from different worlds. I have thrown away such prejudice. Now, I try to talk with anyone rather than people whom I like” (Individual, Gaoen 27/06/2014).

In brief, WTC was revealed to have a positive impact on the multilinguals’ L2 Chinese speech performance and production. Such a finding supports Segalowitz’s (2010) L2 speech production model, positing that motivation to communicate could directly influence learners’ L2 speech production. However, our data also showed that WTC could be volitionally controlled. In other words, learners could consciously determine the quality and quantity of their speech production. Teachers, therefore, should understand that speaking less may not necessarily mean less proficiency, but rather, learners volitionally do not want to talk much about the topic. In order to enhance learners’ L2 WTC, teachers may consider conducting a needs analysis to capture learners’ interests in speaking, so that interesting and engaging topics and activities could be designed to better serve classroom teaching (Zarrinabadi, 2014).

### Socio-Cultural Factors Influencing Multilinguals’ L2 Chinese Speech Performance and Production Ability Development

This study revealed that L2 cultural interest, L2 communities, and L2 classes were prominent socio-cultural factors contributing to the multilinguals’ L2 Chinese speech production ability development (see Table 4).

**TABLE 4:** Socio-cultural dimensions of multilinguals’ L2 Chinese speech production.

**Socio-cultural factors**	**Views on contributing factors to L2 speech production**
L2 cultural interest	∙ Positively contributes to L2 speech production ability development, particularly in terms of vocabulary, fluency, and authenticity (17/17)
L2 communities	∙ Positively contributes to L2 speech production ability development through social contacts and interactions (16/17) ∙ Economically developed communities will be more attractive to learners
L2 classes	∙ Positively contributes to L2 speech production ability development (14/17) ∙ Neutral attitude toward Chinese classes (3/17)

#### L2 Cultural Interest

All the 17 L2 Chinese multilinguals pointed out that they were very attracted to Chinese culture, specifically Chinese movies and television. Such an attachment contributed to the progress of their L2 Chinese speech production ability, particularly in terms of aspects such as vocabulary, fluency, and authenticity. As Tom pointed out, he liked Chinese culture very much, such as Chinese television, movies, music, and entertaining shows. “Through watching these TV shows, [Tom gets] to know the current situations of China, people’s opinions at different ages, and popular online expressions. All of these help enlarge [his] vocabulary, enhance [his] authenticity in speaking Chinese, and enriches [his] knowledge of Chinese cultural etiquette” (Individual, Tom 29/06/2014). This echoes Dan’s viewpoint: “I really like Chinese movies and television. Dialogues in movies and television sound more authentic compared with textbooks. I find my speaking has improved so much because of watching movies and television” (Individual, Dan 15/06/2014).

#### L2 Communities

The 17 L2 Chinese multilinguals also indicated that their attitudes toward Chinese communities were positively linked with their L2 Chinese speech production ability development. To be more specific, 16 out of the 17 suggested that they liked living and traveling in China and made many friends with local Chinese. As Mads pointed out, “living in China offers me opportunities to communicate and to get to know Chinese across all walks of life, which greatly improved my L2 Chinese speaking ability” (Individual, Mads 23/05/2014). Only one participant suggested that his attitude toward the Chinese community was neutral, pointing out that “[he spends] most of [his] time staying in [his] dorm reading books or surfing the Internet. So, [he is] not sure whether [he likes or dislikes] the Chinese community” (FG, Yeats 25/04/2014). Despite his neutral attitude, he also added: “if you want to speak authentic Chinese, you should appreciate the local culture and make friends with local people” (FG, Yeats 25/04/2014).

However, individuals’ attitude toward a community could be subject to its economic status. High economic status would result in a positive attitude toward the community among L2 learners, and vice versa. Learners with a positive attitude toward a community would have more opportunities and be more willing to socialize with the local people. As a result, learners’ L2 speech production ability could be improved through positive social contacts and interactions. As Gaoen pointed out, “I did not like the local environment and dialects in Guangxi. The learning opportunities there were limited and the learning entertainment was so poor. My Chinese did not improve much there” (Individual, Gaoen 27/06/2014). Gaoen further claimed, “when you love a city, you will love the language there. You will be more active and willing to communicate with the locals, to go traveling, to get to know every aspect of life, and to adapt yourself to the society” (Individual, Gaoen 27/06/2014).

#### L2 Classes

The majority (14/17) of the multilinguals held a positive attitude toward L2 Chinese classes, believing that L2 Chinese classes contributed much to their speech production ability development. Specifically, 11 respondents pointed out that they liked having classes in school, because “Chinese teachers can anticipate problems that learners may have in class. Moreover, students can get instant help if they have any questions” (FG, Feng 08/06/2014). As a result, “students will become more cooperative in class, which will contribute to the improvement of their L2 speaking ability, such as in terms of grammar, sentence structure, and pronunciation” (FG, Judy 10/06/2014). Three respondents held Chinese classes in high regard. They enjoyed having classes with native Chinese students, “because it is an effective way to improve overall Chinese quickly” (FG, Krimu 08/06/2014).

Nevertheless, the rest (3/17) held a neutral attitude toward Chinese classes, pointing out that “the knowledge from class is limited, because a lot of authentic expressions and slangs cannot be found in textbooks” (FG, Jenny 10/06/2014). Moreover, “taking only Chinese language courses was slightly boring while taking major courses was slightly too difficult. It is hard to find the balance” (Individual, Dan 15/06/2014).

#### Summing up

As suggested from the above, almost all the multilinguals in this study held positive attitudes toward L2 Chinese culture, community, and classes. These positive attitudes were found to be beneficial to the development of their L2 Chinese speech production ability. Such a finding adds more evidence to the literature from a socio-cultural perspective that L2 learning enjoyment could significantly impact learners’ L2 learning success (Dewaele and Li, 2018; Jin and Zhang, 2018, 2019; Li et al., 2019). Specifically, learners with strong interests in culture, society, and language would be more willing to be exposed to interactive social environments. As a result, knowledge and experiences derived from such intensive language contact contexts could sustain and facilitate learners’ further improvement of their L2 speech production ability (Amiryousefi, 2018; Minagawa et al., 2019). Such a finding also corroborates Segalowitz’s (2010) L2 speech production system, suggesting that language experiences and social contexts could exert a certain influence on learners’ L2 speech production. As a result, teachers may consider incorporating culturally rich, interesting, and engaging activities into classroom teaching so as to establish a positive attitude toward Chinese society, culture, and classes among learners. In addition, learners should learn to appreciate and embrace different societies and cultures so that they will form an inclusive and positive socio-cultural attitude toward L2 learning.

### Influence of Multilingualism on L2 Chinese Speech Performance and Production Ability Development

Apart from the cognitive, affective, and socio-cultural dimensions, multilingualism was also found, to some extent, to contribute to the participants’ L2 Chinese speech performance and production ability development. In general, the impact of multilingualism depends on language distance. Specifically, the closer the distance between different L2s is, the easier the language transfer will be. As June pointed out,

Chinese tones are very difficult for most L2 Chinese learners. I found it difficult too. However, compared with other learners from the US and the UK, I have certain advantage. In our mother tongue Burmese, we have four tones as well. Of course, Burmese tones and Chinese tones can be different. At least, I have a tonal sense helping me understand different tones in Chinese. I guess that is why my spoken Chinese does not sound so foreign-like (FG, June 12/04/2014).

It can be suggested from the above that a multilingual background, to some degree, could be conducive to L2 Chinese speech performance and production ability development, particularly when there is a positive multilingual transfer. However, multilingual transfer cannot always be positive in circumstances when there is a fundamental difference between different languages. As Bai pointed out, “Russian is somehow easier for me, although Chinese and Russian are both drastically different from Mongolian. As a Mongolian, I found we have borrowed more words from Russian than Chinese. Also, Mongolian and Russian both use Cyrillic alphabets” (FG, Bai 10/06/2014).

Although there is no direct evidence from our data showing how multilingualism contributes to L2 Chinese speech performance and production ability development, the potential impact of multilingualism should not be overlooked, as evidenced by the literature that multilinguals can be cognitively, affectively, and socio-culturally more at an advantage in learning L2s (e.g., Phongsa et al., 2018; Bright et al., 2019; Cenoz and Gorter, 2019). Nevertheless, teachers and learners should understand that multilingualism does not necessarily guarantee the success of any L2 learning. Rather, a comprehensive diagnosis of L2 learning from cognitive, affective, and socio-cultural perspectives should be performed to fully capture learners’ L2 strengths and weaknesses. As a result, tailored actions can be taken for better learning outcomes.

## Conclusion and Limitation

This study was set up to investigate a group of multilinguals’ perceptions toward their L2 Chinese speech performance and production ability development from the cognitive, affective, and socio-cultural perspectives through an in-depth qualitative inquiry that used focus groups and semi-structured interviews as data collection tools. The results of this study showed that the multilinguals’ L2 Chinese speech performance and production ability development could be the result of the synergistic effects of the cognitive (e.g., age of acquisition, cognitive fluency, learning styles, and speaking strategies), affective (e.g., motivation, anxiety, speaking self-efficacy, and WTC), and socio-cultural (e.g., attitudes toward L2 Chinese culture, community, and classes) dimensions of L2 Chinese learning. In addition, it was also suggested that multilingualism might have a certain influence on multilinguals’ L2 Chinese speech performance and production ability development.

This comprehensive and systematic understanding of multilinguals’ L2 Chinese speech performance and the development of such an ability provides empirical support for Segalowitz’s (2010) L2 speech production model. Specifically, this study enriches the affective dimension of Segalowitz’s (2010) L2 speech production system by pointing out that anxiety and speaking self-efficacy could also contribute to learners’ L2 speech production apart from their motivation to communicate. Moreover, this study offers insights for teachers and learners into how to support learners’ L2 Chinese speech production ability by concurrently taking cognitive, affective, and socio-cultural dimensions into full consideration.

Like many other studies, our study is not exempt from imitations. First, this study was only a cross-sectional qualitative study that investigated multilinguals’ perceptions of L2 Chinese speaking. Future studies may consider adopting other qualitative methods, such as retrospective diaries and think-aloud protocols, to capture multilinguals’ perceptions from a longitudinal perspective. Also, future research may want to make use of a mixed-methods or quantitative approach to strengthen the trustworthiness of the findings. Second, although this study supports the literature that learners’ multilingual background could influence L2 learning, how multilingualism interplays with cognitive, affective, and socio-cultural factors in influencing multilinguals’ L2 Chinese speech performance and production ability development was not examined. Future research may consider taking the influence of multilingualism into more detailed account. Last, the multilinguals’ L2 Chinese speaking was self-diagnosed, which may not accurately reflect learners’ genuine language proficiency. Alternatively, tests could be developed to measure learners’ actual L2 speech production ability in future research.

## Data Availability Statement

The datasets for this article are not publicly available. Requests to access the datasets should be directed to the first author.

## Ethics Statement

The studies involving human participants were reviewed and approved by the University of Auckland. The patients/participants provided their written informed consent to participate in this study. Written informed consent was obtained from the individual(s) for the publication of any potentially identifiable images or data included in this article.

## Author Contributions

PS and LZ conceived and designed the study, and proofread and revised the manuscript. PS collected the data and drafted the manuscript.

## Conflict of Interest

The authors declare that the research was conducted in the absence of any commercial or financial relationships that could be construed as a potential conflict of interest.

## Appendix

### Semi-Structured Interview Prompts (Sample Version)

• What do you think of your L2 Chinese speech production and such an ability?
• What motivates you to speak L2 Chinese well or to improve your L2 Chinese speech production ability?
• What makes you feel unconfident when speaking Chinese?
• What would you do to strengthen your confidence in speaking?
• Do you feel nervous when you are speaking Chinese? Why or why not?
• Do you have any moments when you feel that you can totally control your speaking?
• Under what circumstances will you be more willing to speak Chinese?
• Do you use any speaking strategies when speaking Chinese? Any strategies that you use to improve your L2 Chinese speech production ability?
• Do you think your speaking strategies have anything to do with your L2 Chinese speech production and the development of such an ability?
• Do you agree with that the younger you started learning Chinese, the better your L2 Chinese speech production and/or ability would be?
• What is your learning style like?
• Do you think your learning styles have anything to do with your L2 Chinese speech production and the development of such an ability?
• Do you like living in China? If yes, do you think living in China has anything to do with your L2 Chinese speech production and the development of such an ability?
• Do you like Chinese culture? If yes, do you think Chinese culture has anything to do with your L2 Chinese speech production and the development of such an ability?
• Do you like Chinese classes? If yes, do you think Chinese classes that you took have anything to do with your L2 Chinese speech production and the development of such an ability?
